# Assessing reflective functioning in prospective adoptive parents

**DOI:** 10.1371/journal.pone.0245852

**Published:** 2021-01-26

**Authors:** Saskia Malcorps, Nicole Vliegen, Liesbet Nijssens, Eileen Tang, Sara Casalin, Arietta Slade, Patrick Luyten

**Affiliations:** 1 Faculty of Psychology and Educational Sciences, KU Leuven, Leuven, Belgium; 2 Faculty of Psychology and Educational Sciences, Vrije Universiteit Brussel, Brussels, Belgium; 3 Yale Child Study Center, Yale University, New Haven, Connecticut, United States of America; 4 Research Department of Clinical, Educational and Health Psychology, University College London, London, United Kingdom; International Telematic University Uninettuno, ITALY

## Abstract

The capacity for reflective functioning (RF) or mentalizing of adoptive parents is hypothesized to play an important role in fostering socio-emotional development in adopted children. This paper reports on the development and preliminary validation of the Adoption Expectations Interview (AEI), a semi-structured interview to assess RF in prospective adoptive parents. The AEI was developed based on the Pregnancy Interview, Parent Development Interview, and Working Model of the Child Interview, three interviews that have been used to assess RF in biological parents, to capture RF before child arrival in prospective adoptive parents. In a sample of 96 prospective adoptive parents, the Reflective Functioning Scale, as applied to the AEI (AEI-RFS) showed good reliability, with strong correlations between the different demand items, high internal consistency, and good to excellent inter-rater reliability. A principal component analysis yielded one component, suggesting that the items measured a unidimensional factor. Preliminary evidence for the construct validity of the AEI-RFS was demonstrated by significant associations between the AEI-RFS and well-validated measures of mentalizing, attachment dimensions, and interpersonal functioning.

## Introduction

Parental mentalizing, or parental reflective functioning (PRF), refers to the parent’s capacity to think about their child as having an inner world and to treat the child as a psychological agent with his or her own feelings, wishes, and desires, as well as to recognize that the child’s state of mind can influence the parent’s own mental states and behavior [1]. PRF has been shown to play an important role in explaining the intergenerational transmission of attachment and the socio-emotional development of children more generally [2–4, for a meta-analysis, see 5]. Moreover, recent studies suggest that high levels of PRF may be an important protective factor, particularly among children who grow up in contexts of psychological or social risk [6–8].

In this context, several authors have suggested that the reflective functioning (RF) of adoptive parents may be of particular importance in fostering the socio-emotional development of adopted children, as a large subgroup of these children have a history characterized by substantial trauma, in particular, attachment trauma [9–11]. Research by Miriam and Howard Steele and colleagues, in particular, has been instrumental in suggesting that both pre-adoptive and post-adoptive *parental states of mind* might be an important protective factor in the development of child attachment representations in the months and years after adoption [12, 13].

Given these encouraging findings, further research on the putative role of PRF in the development of adopted children is needed. However, research in this area is hampered because there are currently no measures specifically designed to assess RF in prospective adoptive parents. In this paper, we report on the development and preliminary validation of the Reflective Functioning Scale (RFS) [14] as rated on the Adoption Expectations Interview (AEI) [15], a semi-structured interview adapted from three measures that have been typically used to assess RF in (prospective) parents: the Pregnancy Interview (PI) [16], the Parent Development Interview [17] as adapted for adoptive parents [18], and the Working Model of the Child Interview (WMCI) [19]. We first discuss the purported role of PRF and prenatal RF in biological and adoptive families. Next, we describe the RFS, followed by the rationale and aims of the present study.

### The role of PRF in biological and adoptive families

In the past decade, research regarding PRF in biological families has convincingly demonstrated that higher levels of PRF are prospectively related to better child social outcomes and less internalizing and externalizing problems [20, 21]. In addition, recent meta-analyses showed robust associations between PRF, parent–child attachment, parent sensitivity [5] and child mentalizing [22].

As noted, PRF may play an important role in mitigating the effects of early adversity [6–8, 23] and negative parenting behaviors more generally [24, 25] on children’s socio-emotional development. Studies have shown that among children who grow up in adverse circumstances, those whose parents have higher levels of PRF demonstrate better social functioning and mentalizing abilities, more secure attachment patterns, and fewer externalizing problems than those whose parents have lower PRF. Moreover, interventions in at-risk families (e.g. families living below the poverty line with limited education and other resources) that focus on improving RF have been shown to positively influence a wide range of parent and child outcomes, such as PRF, parental pathology, child physical and socio-emotional development, and child attachment in particular [26–28].

As adopted children have frequently experienced a variety of adversities in early development, (e.g. separation of their biological parents, inadequate care from early caregivers or orphanages, abuse, and/or disruptive changes of context and culture), PRF may be relevant in buffering the effects of this early adversity [11]. Although scarce, the studies conducted to date with adoptive families indeed show that a more positive and reflective parental stance towards the adopted child is associated with positive child outcomes such as less aggressive themes in children’s narratives of attachment-related story stem tasks [18], less child negative demeanor in a parent-child co-construction task [29], and fewer child behavioral difficulties [30, 31]. Furthermore, in recent years various PRF-based programs for foster and adoptive parents have been developed and implemented. Preliminary results suggest that this PRF-based approach helps to contain family problems, reduces parenting stress, and increases PRF and the parents’ understanding of their children’s behavioral difficulties [9–11].

It must be noted that the majority of studies in this area have focused on maternal PRF. To date, there is a paucity of research on paternal PRF and the potential differential impact of maternal and paternal PRF on child development. While some of these studies have found that high levels of maternal and paternal PRF have a similar positive influence on important aspects of child development such as early attachment security [3], child competences and socio-emotional problems [32], and adolescent RF [24], others have found that maternal, but not paternal, RF during pregnancy was associated with Theory of Mind performance at age 5 [33], while another study reported that paternal, but not maternal, PRF correlated with the social competence of adolescent children [24]. More research concerning the respective roles of maternal and paternal PRF is therefore clearly needed [23].

### The importance of prenatal RF

In biological families, there is also evidence that prenatal RF, that is, the parent’s ability to think of the fetus as a separate individual with developing personal features, needs, and temperament [34], supports the early parent–child relationship and helps parents to cope with the often stressful transition into parenthood. In several studies, prenatal RF was associated with higher parental sensitivity, attachment security, PRF, and positive engagement [3, 35–38]. Furthermore, higher levels of RF during pregnancy or just after childbirth are related to (a) better interpersonal adjustment, with higher levels of co-parenting quality and dyadic cohesion with the partner [39], (b) lower levels of attachment anxiety and avoidance [40], and (c) lower alexithymia [41].

In adoptive families, the potential role of parents’ pre-adoptive PRF in explaining the socio-emotional development of adopted children has not yet been investigated. This is surprising as, for instance, in Flanders (Belgium), PRF is a central characteristic that is screened for in the process of social enquiry that parents have to undergo before receiving juridical permission to adopt. Yet, to date we do not know how these abilities affect the adopted child’s development in later life. However, studies by Steele and colleagues demonstrated the importance of such pre-adoptive parental states of mind. In the Attachment Representations and Adoption Outcome Study, they found that the attachment representations of prospective adoptive parents, measured pre-placement, were associated with the parent’s emerging child and parenting representations and the child’s attachment stories as measured by an Attachment-Story Stem Completion Task 3 months after placement [12, 18]. Moreover, while all children showed an increase in positive themes in their attachment stories after 2 years in the adoptive families, the few children who also showed a decrease of negative themes were more likely to have adoptive parents whose Adult Attachment Interviews (AAIs) pre-placement were judged to be secure-autonomous [13, 42].

### The Reflective Functioning Scale

Various measures have been developed to assess RF [43], of which one of the most important remains the RFS, which is typically scored on detailed accounts of past and current attachment experiences based on semi-structured interviews with participants.

The RFS ranges from negative (score –1) to exceptional or full (score 9) RF; scores from –1 to 3 are considered to be negative to low, scores between 5 and 9 are considered to reflect average to high RF, and a score of 4 is referred to as borderline [44]. Coding the RFS follows a standardized procedure [14] that is based on the presence and sophistication of four domains of qualitative markers of RF within responses to every interview question, namely: (a) awareness of the nature of mental states (e.g. opaqueness of mental states), (b) explicit efforts to tease out mental states underlying behavior (e.g. causal accounts of behavior in light of mental states), (c) recognizing developmental aspects of mental states (e.g. recognition that mental states can change over time), and (d) mental states in relation to the interviewer (e.g. acknowledging separateness of minds).

Over the past few decades, studies have supported the reliability and validity of the RFS using interviews that focus on (a) past attachment experiences with one’s own caregivers as rated on the AAI [44, 45], (b) the current relationship with a specific biological or adopted child using the PDI [1, 18, 46] and the WMCI [47], and (c) prospective parents’ expectations as assessed during pregnancy using the PI [35, 36, 48].

As these interviews focus on biological parenthood and/or the pregnancy that preceded it, they are often inadequate to appraise experiences from adoptive parenthood or the path leading up to it. In order to assess PRF and associated concepts in adoptive parents, Miriam and Howard Steele and colleagues adapted the PDI and its coding manual to accommodate the unique features of adoptive families [18] with questions about, for example, the first meeting with the adopted child, moments where parents did or did not feel that they ‘clicked’ with their child, and scoring categories concerning the level of social support experienced by the parents, and feelings of disappointment/despair about the new relationship. A factor analysis on the 31 PDI rating scales they developed led to the identification of three overarching dimensions: positive/reflective parenting, negative/angry parenting, and despair [18].

While this specifically tailored PDI allows the assessment of post-adoption RF, currently there is no comparable instrument to measure pre-adoption RF. There is therefore a need for a narrative measure of PRF in prospective adoptive parents.

### The present study

This paper reports on the development and preliminary validation of the Adoption Expectations Interview (AEI) in a sample of 96 prospective adoptive parents (*N* = 48 couples). The AEI is a semi-structured interview that is specifically tailored to experiences of the adoption process and expectations about upcoming adoptive parenthood, and is designed to assess pre-adoptive RF in prospective adoptive parents (referred to as AEI-RFS). The AEI was adapted based on the PI, PDI, and WMCI. The first aim of this study was to investigate the reliability of the AEI-RFS by examining correlations between the different RF demand items and the RF global score, the factor structure and internal consistency of the different demand items, and the inter-rater reliability of the RF global score and the RF demand items. We expected moderate to strong relations (a) between the different RF demand items and (b) between the RF demand items and the RF global score [46]. In addition, inter-rater reliability was examined. Next, based on the studies of Taubner et al. [44] and Sleed, Slade & Fonagy [46] regarding the psychometric properties of the AAI-RFS and PDI-RFS, we used principal component analysis (PCA) on the demand items of the AEI to investigate whether one unidimensional factor underlay these items.

Second, we investigated the relation between the AEI-RFS and demographic features. In Flanders, the suitability of prospective parents to adopt is investigated in a social enquiry that is commissioned by a youth court; this process includes a home visit by a social worker and several interviews with a social worker and a psychologist. Additionally, prospective parents have to complete a 3-day preparation course offered by the Flemish Center for Adoption. The complete adoption process (social enquiry and waiting time) takes several years. As a result, adoptive parents in Flanders are a relatively homogenous group in terms of age, psychological functioning and educational background. Therefore, we expected that the AEI-RFS global score would not be associated with age or level of education.

In line with the evidence on sex differences in RF [49], we expected that prospective adoptive mothers would demonstrate higher levels of RF than fathers. Furthermore, given that all participants went through an extensive psychosocial screening procedure as part of the adoption process, we expected to observe relatively high RF scores and little variability within the sample. Specifically, we predicted that the number of parents showing negative to low mentalizing (score <4 on the RFS) would be limited.

Finally, we evaluated the construct validity of the AEI-RFS by exploring the associations of the AEI-RFS with self-report questionnaires regarding mentalizing and related constructs, such as attachment dimensions and interpersonal problems.

## Materials and methods

### Participants and procedures

This study used data from the Leuven Adoption Study (LAS), a multi-wave, multi-method, and multi-informant study about the development of internationally adopted children in Flanders (Belgium) which was approved by the Social and Societal Ethics Committee of the University of Leuven (KU Leuven) in 2008 and 2013. Recruitment for the LAS was done through adoption agencies, social media, and meetings of prospective adoptive parents. Couples who were interested in participating in the study first received a leaflet giving detailed information about the study. If they wanted to participate, they met with a research assistant who provided further detailed information about the study. After participants provided written informed consent, a home visit was scheduled to take place before the arrival of the adopted child. During this visit, the AEI was administered to parents, and parents were also asked to complete a booklet of questionnaires and to return it within 3 weeks of receipt. Up to five reminders were provided by e-mail or telephone (Time 1).

Inclusion criteria were (a) parents in a heterosexual relationship who (b) had no biological children of their own, (c) had applied for the international adoption of a first child, (d) and had received official permission to adopt from the Belgian Central Authority for adoption. Exclusion criteria were (a) if the adopted child was older than 2.5 years at arrival and, (b) if the parents decided to not raise the adopted child in the Dutch language.

Participants were 48 heterosexual couples (96 parents) who at the start of the study were on the waiting list of a specific adoption agency. All couples had Belgian nationality and spoke Dutch as their first language. The couples were all married and the duration of their relationships ranged from 4.25 to 20.41 years (*M* = 10.83, *SD* = 3.56). Information regarding participants’ age and level of education is provided in Table 1.

**Table 1:** Demographic features of participants.

Variables	Fathers	Mothers
**Age (*M* (*SD*)) in years**	34.29 (3.80)	33.10 (3.32)
**Age (range) in years**	27–46	27–42
**Highest obtained degree**		
** High school**	29.17%	12.5%
** Higher education**	70.83%	87.5%
** • Bachelor’s**	35.72%	47.92%
** • Master’s**	35.11%	39.58%

Note. N = 48 couples, *M* = Mean, *SD* = Standard Deviation.

### Instruments

#### Adoption expectations interview

The AEI [15] is a semi-structured interview rooted in contemporary attachment theory, and is largely based on the PI developed by Arietta Slade and colleagues [16]. In addition, it draws on other interviews aimed at assessing parents’ internal working models or mental representations of parents, such as the PDI and WMCI. The interview is also influenced by research on PRF and contains several questions and probes that are aimed at explicitly assessing this capacity [similar to the adaptation of the WMCI to the RFS; 47], for example, “*How did their reaction make you feel*?”, “*Why do you think they reacted that way*?”.

The AEI consists of 20 questions that aim to assess the expectations of prospective adoptive parents with regard to adoption and their future adopted child in particular, with a focus on the internal working models or mental representations adoptive parents have of their future adopted child. The interview invites parents to look forward (e.g. to the needs of the child in the period after arrival, to the future parenting role of self and partner) and also to look back and reflect on their experiences from their initial decision to try to get pregnant to their decision to adopt a child, and the extensive psychosocial screening process preceding adoption, to the day they received the news that they had been assigned a child to adopt.

The AEI taps into different domains of interest. The first six questions aim to assess the feelings about the adoption and the adoption process of the interviewee him/herself, as well as his/her partner and his/her context (family, friends, etc.). For instance: “*Can you remember the moment you first thought about adoption and how this led to the decision to adopt a child*? *(a) Tell me about that moment*. *(b) How did you feel*? *(c) Why do you think you reacted that way*?”

A second group of questions (questions 7–13) are more focused on the child—that is, the current fantasies and feelings about the child, the expected care the child will need, and the imagined impact the child will have on the parent. An example question of this is: “*Would you say you have a relationship with the adoptive child now*? *If they say yes*, *can you think of two words to describe that relationship*? *If they say no*: *why not*? *Can you tell me a little bit more about that*?”

A third group of questions (questions 14–17) focuses on the impact the adoption process has had on the interviewee’s relationship with his/her partner and parents, and how they imagine their own style of parenting will be similar to or different from their parents’. For instance: “*In what ways do you imagine you will be like your mother/father as a parent*?”

Question 18 asks the interviewee to look back on the moment he or she first wanted a child, while questions 19 and 20 focus on how prospective adoptive parents imagine their future life will look like (e.g., “*If you could take a leap in time and fast forward 5 years*, *which three wishes would you at that point have for your child*?”).

In this study the AEI was conducted individually with each parent by a research assistant during a home visit. The duration of one interview was on average approximately 1.5 hours. Interviews were later transcribed verbatim by research assistants who were not involved in conducting or scoring the interviews.

#### The Reflective Functioning Scale as coded on the AEI

First, three of the authors (SM, LN, and PL) categorized the AEI questions as “demand” or “permit” questions. Demand questions probe for RF by asking about mental states explicitly (e.g., “*How did that make you feel*?”), and permit questions allow interviewees to demonstrate RF but do not probe for it [44]. This distinction is important for later coding because while answers on demand questions always receive a score, permit questions will not be penalized for absence of RF (score 1–3).

Second, one of the authors (SM) scored the complete AEI of 10 randomly selected interviews using the RFS to compare the level of RF as scored on the complete interview (including all demand and permit questions) versus RF based on scoring only the AEI demand questions. Given that the discrepancy between the two scoring methods was .50 points on the RF global score at most, and other shortened versions of narrative methods to score RF have shown promising results [8, 50, 51], we decided to score the AEIs based on the demand questions only. However, raters read the complete interview (including all permit questions) when scoring so as to have access to all necessary contextual information and to make sure that certain mental-state attributions were not more thoroughly explained in previous questions.

Third, inter-rater reliability between two of the authors (SM and LN) was determined based on a set of 10 randomly selected interviews. Both researchers were trained in RFS scoring on the AAI or PDI. Given the high inter-rater agreement (see below), both raters then each coded half of the remaining interviews separately.

#### Experiences of close relationships–revised

Attachment in romantic relationships was assessed using the Experiences of Close Relationships–Revised [ECR-R; 52], which contains 36 items and consists of two subscales of 18 items: Attachment Anxiety (ECR-Anx, e.g., “*I often worry that my partner will not want to stay with me*”) and Attachment Avoidance (ECR-Avo, e.g., “*I get uncomfortable when a romantic partner wants to be very close*”). Each item is scored on a seven-point scale ranging from 1 (*strongly disagree*) to 7 (*strongly agree*). Previous studies showed clear evidence for the two-factor structure, temporal stability, and construct, discriminant and convergent validity of the ECR-R [53]. In the current study, Cronbach’s α of ECR-Anx was acceptable (α = .73), and that of the ECR-Avo subscale was low (α = .43). In order to increase internal consistency, we removed four items of the ECR-Avo subscale, which led to acceptable to good internal consistency (Cronbach’s α = .76).

#### Reflective functioning questionnaire

The Reflective Functioning Questionnaire [RFQ, 54] is an eight-item self-report questionnaire that measures the level of certainty (RFQ-C) and uncertainty (RFQ-U) about mental states. All items are scored on a 7-point Likert scale, ranging from “*completely disagree*” to “*completely agree*”. Higher scores on the RFQ-C reflect more certainty and, in the extreme, hypermentalizing (the over-attribution of mental states to others that goes far beyond the relevant or observable context) [55], while high scores on the RFQ-U reflect a stance characterized by an almost complete lack of knowledge about mental states. Satisfactory reliability, construct and convergent validity of the RFQ was previously demonstrated in adult clinical and non-clinical populations [54, 56]. In the current study, the internal consistency of the RFQ-U and RFQ-C scales was acceptable, with Cronbach’s α = .61 and .73, respectively.

#### Toronto Alexithymia Scale

The Toronto Alexithymia Scale [TAS-20; 57] is a self-report measure containing 20 items that are rated on a 5-point Likert scale, ranging from “*completely disagree*” to “*completely agree*”. The TAS-20 consists of three subscales assessing difficulties in identifying feelings (DIF; e.g., “*I am often confused about what emotion I am feeling*”) and difficulties describing feelings (DDF; e.g., “*It is difficult for me to find the right words for my feelings*”), and an externally oriented thinking style (EOT; e.g., “*I prefer to just let things happen rather than to understand why they turned out that way*”). For this study we used the TAS-Total Score, which is a sum score of the subscales. A recent review of the TAS-20 indicated “good” internal consistency of the Total Score, “good-to-excellent” test–retest reliability, and strong evidence of the convergent validity of the scale [58]. In the present study, the internal consistency of the TAS-20 was good, with Cronbach’s α = .85.

#### Inventory of interpersonal problems

The Inventory of Interpersonal Problems [IIP-32; 59] is a self-report measure that assesses interpersonal problems and includes eight different subscales, each comprising four items: (a) Domineering/Controlling, (b) Vindictive/Self-Centered, (c) Cold/Distant, (d) Socially Inhibited/Avoidant, (e) Non-Assertive, (f) Overly Accommodating, (g) Self-Sacrificing, and (h) Intrusive/Needy. The IIP-32 includes items regarding interpersonal behaviors, which participants identify as “*hard to do*” or “*does too much*” on a 0 (*not at all*) to 4 (*extremely*) Likert scale. Psychometric studies have indicated acceptable to good internal consistency and concurrent validity of both the Dutch and English versions of the IIP-32 [60, 61]. For this study, the mean total score of the 32 items was used as a measure of the general level of interpersonal problems. Internal consistency of the total scale was excellent, with Cronbach’s α = .90.

### Analyses

First, we calculated Pearson correlations between the RF demand items and the RF global score. Second, the inter-rater reliability of two raters (SM and LN) was assessed on a subset of 10 interviews by determining the intraclass correlations (ICCs) for absolute agreement using a two-mixed model for both the RF global score and the different RF demand items. To express the agreement between both raters we used guidelines proposed by Cicchetti [62], with ICCs < .40 considered to reflect poor agreement, between .40 and .59 fair agreement, between .60 and .74 good agreement, and between .75 and 1 excellent agreement. Third, the internal consistency of the RF demand items was determined by calculating Cronbach’s α. Next, we conducted a PCA on the eight RF demand items of the AEI. Finally, we investigated the construct validity of the AEI-RFS by calculating Pearson correlations between the AEI-RFS global score and two other measures of mentalizing capacity (TAS-20 and RFQ), and two measures of attachment and interpersonal functioning (ECR-R and IIP-32).

All statistical analyses were performed using SPSS software version 25.0 (IBM, Armonk, NY, USA).

## Results

### Reliability

ICCs (Table 2) of the RF global score and all demand items were excellent (range .703–1). As shown in Table 3, the different RF demand items showed significant correlations (range *r* = .391 to .718, all *p*s < .01). The RF global score was also significantly associated with all the RF demand items (*r*s ranging between .619 and .857, all *p*s < .01). Internal consistency of the RF demand items was excellent, with Cronbach’s α = .91.

**Table 2:** Intraclass correlations for RF global score and RF demand items.

AEI-RFS	ICC
**AEI-RF global score**	.947
**AEI-RF 1**	.942
**AEI-RF 2**	.875
**AEI-RF 3**	1
**AEI-RF 4**	.887
**AEI-RF 5**	.897
**AEI-RF 6**	.848
**AEI-RF 9**	.703
**AEI-RF 18**	.863

Note. AEI-RFS = Reflective Functioning Scale as scored on the Adoption Expectations Interview, ICC = Intraclass Correlation.

**Table 3:** Pearson correlations of RF demand items and RF global score.

	AEI-RF global score	AEI-RF 1	AEI-RF 2	AEI-RF 3	AEI-RF 4	AEI-RF 5	AEI-RF 6	AEI-RF 9
**AEI-RF 1**	.619**							
**AEI-RF 2**	.729**	.545**						
**AEI-RF 3**	.778**	.615**	.619**					
**AEI-RF 4**	.857**	.470**	.538**	.638**				
**AEI-RF 5**	.830**	.456**	.521**	.560**	.771**			
**AEI-RF 6**	.659**	.458**	.391**	.633**	.573**	.551**		
**AEI-RF 9**	.702**	.483**	.530**	.582**	.563**	.520**	.502**	
**AEI-RF 18**	.800**	.403**	.532**	.571**	.718**	.633**	.566**	.595**

Note. AEI-RF = Reflective Functioning Scale as scored on the Adoption Expectations Interview

* *p* < .05

** *p* < .01.

Next, a PCA was conducted on the eight demand items of the AEI. The Kaiser–Meyer–Olkin (KMO) measure verified the sampling adequacy for the analysis, KMO = .90 (“superb” according to Field [63]), and Bartlett’s test of sphericity, χ^2^ (28) = 382,732, *p* = .000, indicated that correlations between items were sufficiently large for PCA. An initial analysis of the eigenvalues revealed that only one component had an eigenvalue over Kaiser’s criterion of 1 (eigenvalue = 4.92), and this component explained 61.49% of the variance. The scree plot also indicated one component, suggesting that the scale was unidimensional (see Table 4).

**Table 4:** Factor loading of the RF demand items.

RF Demand item	Factor Loading
**AEI-RF 4**	.851
**AEI-RF 3**	.828
**AEI-RF 18**	.819
**AEI-RF 5**	.810
**AEI-RF 9**	.760
**AEI-RF 6**	.751
**AEI-RF 2**	.730
**AEI-RF 1**	.715

Note. RF = Reflective Functioning, AEI = Adoption Expectations Interview.

### Validity

The average RF global score was 5.51 (*SD* = 1.01), suggesting that parents showed, on average, “ordinary” RF [14]. While the RF global score was not associated with the participants’ age (*r* = .027, *p* = .794) or level of education (*r* = .156, *p* = .131), there was a significant sex difference: Mothers (*M* = 5.75, *SD* = 1.03) demonstrated significantly higher levels of RF than fathers (*M* = 5.28, *SD* = 1.36, *t* = –2.11, *p* = .04), although the absolute difference was small. This was also reflected in the fact that there were no significant differences in the distribution of RF scores between mothers and fathers (Levene’s test for equality of variances: *F*(1,93) = .809, *p* = .371).

The AEI-RFS global score correlated as expected with self-report measures of mentalizing, interpersonal problems, and attachment anxiety (see Table 5). Higher RF global scores were associated with less pronounced problems in identifying and describing feelings, more certainty and less uncertainty about mental states, fewer interpersonal problems, and less attachment anxiety. However, the AEI-RFS global score was not correlated with attachment avoidance (*r* = .164, *p* = .113). A follow-up analysis showed that in fathers there was a strong positive correlation between AEI-RFS and attachment avoidance (*r* = .474, *p* < .01), while in mothers there was a trend for a negative relationship between AEI-RFS and attachment avoidance (*r* = –.250, *p* = .087).

**Table 5:** Zero-order correlations between AEI-RFS and self-report measures of mentalizing, interpersonal problems, and attachment.

	2.	3.	4.	5.	6.	7.
**1. AEI-RFS**	.209*	–.209*	–.204*	–.274**	–.255*	.164
**2. RFQ-C**		–.718**	–.444**	–.538**	–.339**	.374**
**3. RFQ-U**			.518**	.587**	.280**	–.265**
**4. TAS**				.526**	.420**	–.427**
**5. IIP**					.362**	–.386**
**6. ECR-Anx**						–.551**
**7. ECR-Avo**						1

Note. AEI-RFS = Reflective Functioning Scale as scored on the Adoption Expectations Interview, RFQ-C = Reflective Functioning Questionnaire–Certainty Subscale, RFQ-U = Reflective Functioning Questionnaire–Uncertainty Subscale, TAS = Toronto Alexithymia Scale–Total Score, IIP = Inventory of Interpersonal Problems–Total Score, ECR-Anx = Experiences of Close Relationships–Attachment Anxiety Subscale, ECR-Avo = Experiences of Close Relationships–Attachment Avoidance Subscale

* *p* < .05

** *p* < .01.

## Discussion

We report on the development and preliminary validation of the AEI [15], an interview that aims to assess RF in prospective adoptive parents. The AEI, which is rooted in attachment and mentalizing approaches, was based on similar interviews for parents of biological children.

The RFS scores of the eight RF demand items had good reliability, as indicated by their high internal consistency and high correlations between the different RF demand items. Furthermore, a PCA suggested a one-factor solution. These findings are consistent with other studies reporting that a single factor best represents the structure of the RF demand items on the AAI and the PDI [44, 46]. In this context, it should be noted that some studies found a two-factor structure for the RFS as scored on the PDI [64, 65], with separate factors for self mentalizing and child mentalizing. However, as the AEI does not specifically focus on the parent–child relationship, but on other attachment figures and experiences related to the adoption process, we did not expect to find two factors underlying RF in the AEI. The one-factor solution of the AEI is further supported by the high correlations between the RF demand items and the RF global score (*r*s ranging between .62 and .86). Of note, results suggest that raters who have been previously trained on the AAI and the PDI are able to attain excellent inter-rater reliability for both the RF demand items and the RF global score, although further replication of this finding is needed.

As expected, the RF global score did not show associations with age and level of education. However, the global RF score did differ significantly between prospective adoptive mothers and fathers, with mothers demonstrating slightly higher RF scores. This is in line with studies suggesting that women in general perform better on measures of mentalizing. Most likely this sex difference is the result of biological, social and cultural factors. Research suggests that such differences may stem from the different cognitive strategies men and women employ when processing social cognitive information [66]: while men are more disposed to “systemizing” strategies (i.e. the analysis of rule-driven behavior in non-agentive systems), women tend to employ more “empathizing” strategies (i.e. predicting and identifying another’s thoughts and emotions and responding appropriately), which may explain why they perform better on affective-focused mentalizing tasks (e.g. emotion recognition, empathy, social sensitivity) [67]. Furthermore, from a more societal and cultural perspective, men and women are raised quite differently: socialization strategies still encourage the awareness, display and communication of emotions more in girls than in boys, leading to higher levels of mentalizing in girls [49].

Significant and theoretically expected associations with self-report mentalizing measures, attachment, and interpersonal problems provided preliminary support for the construct validity of the AEI-RFS global score. The strength of these associations was modest to moderate, which is consistent with associations of other interview-based RFS measures and self-report questionnaires on mentalizing [68] and interpersonal functioning [69]. Interestingly, post-hoc analyses showed that the relations between attachment avoidance and anxiety and AEI-RFS differed for mothers and fathers. While in mothers high levels of RF tended to be negatively associated with attachment avoidance, in line with theoretical expectations, in fathers high RF seemed to be related to attachment avoidance. This seemingly paradoxical finding can perhaps be explained by the fact that fathers in this study showed relatively low levels of attachment avoidance and relatively high levels of RF. It has been argued that, particularly in higher functioning individuals, as in the present sample, attachment avoidance is associated with a more cognitive and controlled style of functioning that is also an essential component of high levels of RF [70]. Although this finding might reflect more general sex differences in attachment and cognitive style, with men, at least in Western cultures, showing somewhat higher levels of attachment avoidance and a more cognitively oriented information processing style [71, 72], it has been suggested that the RFS may have difficulty distinguishing between genuine high RF and so-called pseudomentalizing [43]. Further studies are needed to investigate these assumptions.

The prospective adoptive parents in this study demonstrated on average a score of 5.5 on the RFS, which is consistent with an “ordinary” level of RF in which interviewees “give convincing indications to have some kind of a model of the mind of attachment figures as well as a model of their own mind which is relatively coherent even if it is simple, and is unlikely to have been solely derived from shared culture rather than from personal experience” [14, p. 45]. Moreover, with the exception of two fathers with a score of 3, no participants showed negative RF (score –1), lack of RF (score 1), or questionable or low RF (score 3). On the contrary, all other participants showed a level of mentalizing that was at or above the “borderline” score of 4, and 23% of parents demonstrated “marked mentalizing” (scores 7–8). These relatively high levels of RF in this sample most probably reflect the extensive screening process for prospective adoptive parents in Belgium, which involves a series of interviews with both parents by social workers and psychologists. The adoption procedure is also quite lengthy in Belgium, typically taking up to 5–6 years, and requires candidate parents to have sufficient financial resources. As a result, successful adoptive parents are typically relatively older and highly educated; in the current sample, almost 70% of fathers and 88% of mothers had obtained a bachelor’s or master’s degree.

This study has a number of strengths and limitations. A first strength is that the study addresses the lack of research on the purported role of RF in adoptive families. Secondly, we assessed pre-adoptive RF in both mothers and fathers, whereas most studies in this field typically focus on mothers only.

Limitations of this study included the relatively small sample size and the cross-sectional design. Future reports will focus on prospective relationships between AEI-RFS and child socio-emotional functioning, and it is hoped that the present paper will encourage other studies in this area.

Despite these limitations, the AEI-RFS may be a promising instrument to investigate the potential role of pre-adoptive RF in explaining developmental trajectories in adopted children and their parents. On a more clinical level, the AEI can help clinicians to explore prospective adoptive parents’ expectations and possible ambivalent feelings about upcoming adoptive parenthood (the joy of having a child vs. the expected challenges of raising an adopted child and the grief of not being able to get pregnant). Assessing AEI-RFS furthermore will provide clinicians with an indication of the parents’ overall ability to reflect both about themselves as prospective parents and about the future adopted child, both of which could become focal points in a possible therapeutic process.

## Supporting information

S1 Protocol Protocol adoption expectations interview. (DOCX)
