# Supplementary material for: Assessing reflective functioning in prospective adoptive parents
Source: PLoS One. 2021 Jan 26;16(1):e0245852. doi: 10.1371/journal.pone.0245852 (PMC7837464; doi:10.1371/journal.pone.0245852)
Supplement: S1 Protocol — (DOCX) [file pone.0245852.s001.docx]

**The Adoption Expectations Interview (AEI)**

*Patrick Luyten^a,b^, Arietta Slade^c,^ Linda Mayes^b^, Nicole Vliegen^a^, Sara Casalin^a^, Stefan Kempke^a^, & Eileen Tang^a^*

^a^ Faculty of Psychology and Educational Sciences, KU Leuven, Leuven, Belgium;

^b^ Yale Child Study Center, Yale University, New Haven, CT, USA;

^c^ City University of New York, NY, USA

*Correspondence Address:*

Patrick Luyten

Faculty of Psychology and Educational Sciences

Tiensestraat 102 pobox 3720

3000 Leuven

patrick.luyten@kuleuven.be

**The Adoption Expectations Interview (AEI)**

*Note: The AEI is a semi-structured interview that includes many probes aimed at elaboration and clarification. Hence, if parents respond spontaneously to questions, that is fine. However, if parents do not respond spontaneously, or if additional clarification is needed, you should use these probes.*

This interview is about the feelings you’ve been having during the adoption process, your feelings and expectations with regard to your adoptive child and about yourself as a future adoptive father/mother. It has just over twenty questions, and usually takes about an hour to finish. Before we get started, could you tell me just a little bit about the circumstances of the adoption, where you’re living, who you’re living with, what’s happening with the father/mother of the baby, are you working or going to school, etc. (Try to sensitively get a little sense of adoption process and the current context, just so that a context is established for the interview. This section shouldn’t take more than three to five minutes – it is just to get background and to have a little warm-up.)

OK, thanks. That helps me get oriented. So, to start,

1. Can you remember the moment you first thought about adoption and how this led to the decision to adopt a child? (Pause to let her/him think.)

- Tell me about that moment…
- How did you feel?
- Why do you think you reacted that way?

2. Can you remember your partner’s feelings towards adoption? (Pause)

- Describe that moment to me…
- How did you feel about his/her feelings?
- Why do you think he/she felt/reacted that way?

3. Can you remember what your family’s reaction was when you told them that you were going to adopt? (Pause)

- Describe that moment to me…
- How did you feel about their reaction?
- Why do you think they reacted that way?

4a. The adoption process is usually a pretty complicated time in terms of feelings, and ups and downs. Let’s start with your good feelings…What are some of the good feelings you’ve had during the adoption process?

4b. If they are able to name feelings, probe for two of them, one at a time. Think of a time when you felt ____... Can you tell me about that time? Why do you think you felt ____?

5a. Have you had any hard or difficult feelings during the adoption process?

5b. If they can name feelings, probe for two of them, one at a time. Think of a time when you felt ____... Can you tell me about that time? Why do you think you felt ____?

5c. Have you had any worries about the adoptive child?

5d. Think of a time when you felt____... Can you tell me about that time? Why do you think you felt____?

**Probe if the participant can’t talk about negative feelings:** If the participant can’t come up with any negative feelings, probe for the following: “Have you had any worries about the child, or about how you’ll manage once the child is here, in terms of money, where to live, getting help, etc.?” (Do they know how they’re going to manage financially, where they’ll live, how they will get help? Are they planning for it? Have they even thought through it?)

5e. What do you do when you have these feelings?

- Is there anyone you can talk to about the feelings that bother you regarding adoption?
- What makes it helpful to talk to that person?

6. When you received the permission to adopt. Can you remember that moment? Tell me about that moment… how did you feel?

7. Would you say you have a relationship with the adoptive child now?

- **If they say yes,** can you think of 2 words to describe that relationship? What makes you say the relationship is _____? (Probe for both)
- **If they say no:** why not? Can you tell me a little bit more about that?

8. Do you know the sex of the baby?

- **if they say yes**: How do you feel about it?
- **If they say no**: Do you have a preference or feelings either way?

9a. What will your adoptive child need from you after its arrival?

9b. How will you feel taking care of those needs?

10. Take a minute to imagine your adoptive child in the future. What kind of person do you imagine your child is going to be? What’s the idea or picture that comes to mind? (Pause)

- Why do you think ____ comes to mind?

11. When you think of the first six months after the arrival of your adoptive child, when do you imagine you’ll be the happiest? (If necessary: Why do you think that is going to be the happiest time?)

12. When you think of the first six months after the arrival of your adoptive child, when do you imagine will be the hardest time? (If necessary: Why do you think that is going to be the hardest time?)

13. Who’s going to help you take care of the adoptive child after its arrival?

- Do you plan to go back to work/school? (And how easy/hard would that be for you?)
- **If you are**, who will be caring for the child?

14. Since you know that you are going to adopt, what has your relationship with your mother/father been like? (Again, keep in mind that if mother uses any general descriptors, like “better”, probe for that and examples.)

15a. In what ways do you imagine you will be *like* your mother/father as a parent?

15b. In what ways do you imagine you’ll be *different*?

16a. Are there things that you’re afraid you’ll do as a parent?

16b. Perhaps things your parents did to you that you’re afraid you’ll do too?

17a. How has your relationship with your partner been affected by the adoption process?

17b. How do you expect him/her to be involved with the adoptive child?

18. How do you look back now at the period since you first wanted to have a child? How do you feel now about this process? (specifically probe for feelings regarding infertility, possible IVF/ICSI treatment, and evaluation by adoption agencies).

19. ***OK, we’re almost done!*** If you had to think of five years from now, and you had three wishes for your adoptive child, what would they be?

20. Is there anything else about what it’s been like to be in this adoption process that you’d like to add?

Check whether you have covered all the questions.
